# Characterizing Vascular Dysfunction in Genetically Modified Mice through the Hyperoxia Model

**DOI:** 10.3390/ijms20092178

**Published:** 2019-05-02

**Authors:** Luis Monteiro Rodrigues, Henrique Nazaré Silva, Hugo Ferreira, Alain-Pierre Gadeau

**Affiliations:** 1 CBIOS—Universidade Lusófona’s Research Center for Biosciences and Health Technologies, Campo Grande, 1749 024 Lisboa, Portugal; henrique.silva@ulusofona.pt; 2 Pharmacological Sciences Department—Universidade de Lisboa, Faculty of Pharmacy, Av Prof Gama Pinto 1649 003 Lisboa, Portugal; 3 IBEB—Biophysics and Biomedical Engineering Institute, Universidade de Lisboa Faculty of Sciences, Campo Grande 1749 016 Lisboa, Portugal; hugoferreira@campus.ul.pt; 4 INSERM U1034, Adaptation cardiovasculaire à l’ischémie, F-33600 Pessac, France; alain.gadeau@inserm.fr

**Keywords:** hyperoxia, mouse, LDF, wavelet transform, diabetes, cardiac hypertrophy

## Abstract

Modelling is essential for a better understanding of microcirculatory pathophysiology. In this study we tested our hyperoxia-mouse model with healthy and non-healthy mice. Animals (*n* = 41) were divided in groups—a control group, with 8 C57/BL6 non-transgenic male mice, a diabetic group (DB), with 8 C57BLKsJ-db/db obese diabetic mice and the corresponding internal controls of 8 age-matched C57BLKsJ-db/+ mice, and a cardiac hypertrophy group (CH), with 9 FVB/NJ cα-MHC-NHE-1 transgenic mice prone to develop cardiac failure and 8 age-matched internal controls. After anesthesia, perfusion data was collected by laser Doppler flowmetry (LDF) during rest (Phase 1), hyperoxia (Phase 2), and recovery (Phase 3) and compared. The LDF wavelet transform components analysis (WA) has shown that cardiorespiratory, myogenic, and endothelial components acted as main markers. In DB group, db/+ animals behave as the Control group, but WA already demonstrated significant differences for myogenic and endothelial components. Noteworthy was the increase of the sympathetic components in the db/db set, as in the cardiac overexpressing NHE1 transgenic animals, reported as a main component of these pathophysiological processes. Our model confirms that flow motion has a universal nature. The LDF component’s WA provides a deeper look into vascular pathophysiology reinforcing the model’s reproducibility, robustness, and discriminative capacities.

## 1. Introduction

The use of oxygen as a challenger has been proposed to study in vivo microcirculatory adaptation in health and disease [1,2,3], even considering the conflicting variability in response to hyperoxia. This variability has been mainly attributed to heterogeneity among studies and protocols. Oxygen exposure time, hyperoxemia cut-off locations [4], assessment timing, and quantification instruments [5] are issues under frequent discussion [5,6].

We recently demonstrated that other factors should be taken into consideration to improve the model and reduce variability, in particular the measurement of the distal effects of hyperoxia in both limbs simultaneously by non-invasive techniques [6].

Laser Doppler perfusion imaging (LDPI) and flowmetry (LDF) are reference systems for these types of studies in human, as in animal models, but these involve some concerns, as both technologies rely on the same biophysical basis, producing the same signal of oscillatory nature, which is very difficult to analyze. Nevertheless, LDF has been used for decades, and also studied with the purpose to find ways to better understand its nature [7]. New mathematical modeling approaches of pulsatile signals, such as LDF, allowed researchers to define time variability, fractal organization, unpredictability, and chaotic profile, among other quantitative variables [8,9]. These modeling approaches have also provided the separation (decomposition) of its main components, meaning that those oscillations, with different time frequencies affecting the signal, can be related to the activity of different tissues or organs [7,10,11,12]. Human LDF components have previously been identified as cardiac, respiratory, myogenic (vascular wall), sympathetic, endothelial NO-dependent and endothelial NO-independent activities [11]. A fast Fourier transform (FFT) or the wavelet analysis (WA) [7,13,14] might easily provide this information, allowing a deeper look into the information included in the LDF raw signal. More sophisticated approaches have been proposed, such as the detrended fluctuation analysis (DFA) to assess its fractal organization and the multiscale entropy analysis (MSE) to observe signal complexity [13].

The present work is in line with our research to improve this methodology, which finds support in a mouse model capable to provide access to vascular pathophysiology by hyperoxia and hyperoxemia [6]. By LDF components analysis in the mouse using the WA, we aim to further characterize vascular dysfunction from different etiologies, trying to bring more discriminative capacities to the hyperoxia model in detecting relevant differences in microvascular function between healthy and non-healthy subjects.

## 2. Results

The O_2_ challenge perfusion response obtained by LDF in both limbs, in all groups, is presented in Table 1, where the expected variability related to hyperoxia is shown. In the C group, all animals respond to hyperoxia with a bilateral perfusion decrease (perD). In DB groups, db/+ animals show a bilateral perD in 7/8 (88%) and a mixed perfusion response (perMIX) in one animal (12%), meaning that one limb shows a decrease of perfusion and the contralateral limb shows the opposite. For db/db animals, a perD is noted in 5/8 (62.5%) animals, while 3/8 (37.5%) show a perMIX response. In CH group, controls (NHE1-WT) 4/8 animals (50%) respond with perD and the remaining 50% with perMIX, while all cardiac overexpressing NHE-1 (NHE1-OE) animals responded with bilateral perD. No matter these differences, relevant statistical differences between limbs’ perfusion could not be found (Table 1), thus, for group comparison we used means from both limbs collected in each phase of the experimental procedure. 

**Figure 1:**
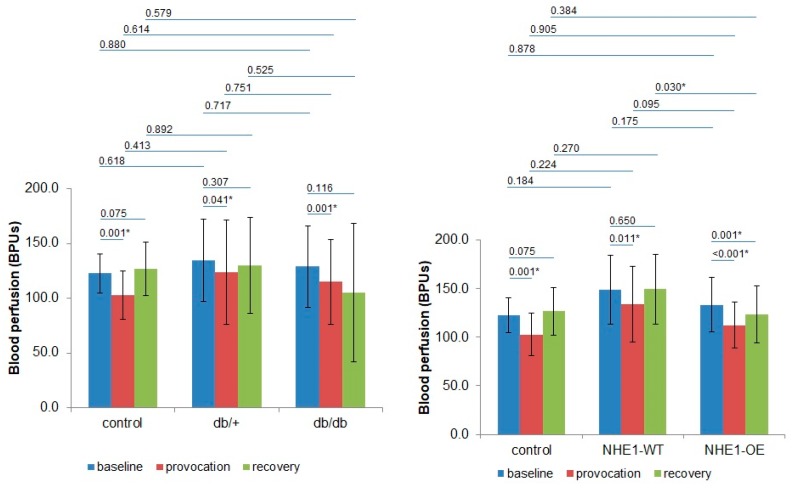
Mean hind limb perfusion changes, expressed in BPUs (blood perfusion arbitrary units) registered in the diabetic (DB) group (**left**, 8 db/db and 8 db/+) and in the cardiac hypertrophy (CH) group (**right**, 8 NHE1-WT and 9 NHE1-OE) versus the Control group (*n* = 8), during the different phases of the experimental protocol. Statistical comparison, and respective *p* values between sub-groups is also shown (m ± sd: mean ± standard deviation; * *p* < 0.05).

**Figure 2:**
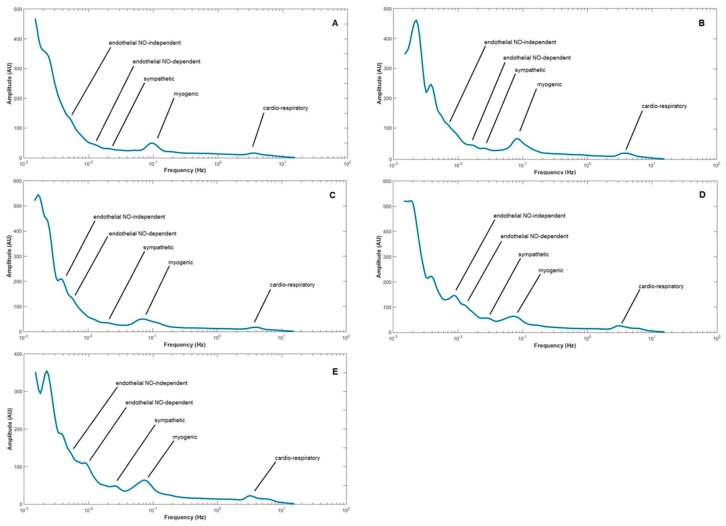
Mean power spectrum of the laser Doppler flowmetry (LDF) signal constructed for each group—Control (**A**, *n* = 8), db/+ (**B**, *n* = 8), db/db (**C**, *n* = 8), NHE1-WT (**D**, *n* = 8) and NHE1-OE (**E**, *n* = 9) from all perfusion mean values obtained during the complete protocol.

Those means are summarized and compared in Figure 1. Hyperoxia significantly decreased perfusion in all groups relative to Phase 1 baseline (Control: 0.001, db/+: 0.041, db/db: 0.001, NHE1-WT: 0.011, NHE1-OE: <0.001). No other relevant differences were detected regarding the Control group or within groups except those in the CH group, where Phase 3 perfusion was still significantly lower than Phase 1 (*p* = 0.001) in NHE1-OE diseased mice group, and a significantly higher perfusion was noted in Phase 3 for the NHE1-WT (*p* = 0.030) compared with NHE1-OE. db/+ and NHE1-WT animals were also compared under these conditions (results not shown) but no differences could be depicted. The application of WA to LDF raw signals reveals the dominant frequencies for each component, where all major band components are defined by their limits (in Hz), and might be represented by 2D frequency spectra (Figure 2). The reference was the human component frequencies [7,11] already used for rats and recently confirmed in the mouse [15]. Our results are remarkably coincident for the low frequency oscillations, e.g., for the endothelial, neurogenic and myogenic components, although some differences arise regarding the cardiac and respiratory components, clearly related with the use of different anesthetic mixtures [15]. The evolution of these bands of dominant frequencies in all groups during our experimental protocol is shown in Table 2.

The cardiorespiratory frequency decreased significantly in all groups during hyperoxia ( Control: *p* = 0.001, db/db: *p* = 0.023, db/+ *p* = 0.001, NHE1-OE: *p* = 0.044, NHE1-WT: 0.031) while the frequency of the myogenic band only decreased significantly in the Control (*p* = 0.002) and db/+ (*p* = 0.023) groups. During recovery, several components also shifted significantly relative to baseline. The cardiorespiratory band showed a significantly higher frequency in the Control (*p* = 0.049), db/db (*p* = 0.003), db/+ (*p* = 0.019), NHE1-OE (*p* = 0.002) groups. The frequency of the myogenic band was higher only in the NHE1-WT wild-type (*p* = 0.041) groups, while for the endothelial nitroxide-dependent (Nod) band, it was higher in the Control (*p* = 0.005) and NHE1-OE (*p* = 0.039). The frequency of the endothelial nitroxide-independent (NOi) band was higher in the Control (*p* = 0.025) and NHE1-WT (*p* = 0.049) groups.

Compared to the Control group, the db/+ mice have significantly lower myogenic frequencies during baseline (*p* = 0.022) and recovery (*p* = 0.019), as did the endothelial NOi during baseline (*p* = 0.038). Similarly, the db/db mice also have significantly lower myogenic frequencies during baseline (*p* = 0.001) and recovery (*p* = 0.003), as the sympathetic (*p* = 0.010) and NOi (*p* = 0.039) during recovery. Comparing the db/+ and db/db groups, the latter have significantly higher cardiorespiratory (*p* = 0.017) and endothelial NOi (*p* = 0.012) frequencies during recovery and baseline. Compared to the Control group, the NHE1-WT mice have a significantly lower cardiorespiratory frequency activity during baseline (*p* = 0.001) and recovery (*p* = 0.001), a significantly higher endothelial NOd (*p* = 0.046) frequency during baseline, and significantly lower myogenic (*p* < 0.001) and endothelial NOi (p = 0.003) frequencies during baseline. Similarly, the NHE1-OE group shows significantly lower cardiorespiratory and myogenic frequencies during baseline (cardiorespiratory: *p* = 0.001; myogenic: *p* = 0.016) and recovery (cardiorespiratory: *p* < 0.001; myogenic: *p* < 0.001) compared to the Control group. Comparing with the NHE1-WT, the NHE1-OE group has significantly lower sympathetic (*p* = 0.035) and endothelial NOd (*p* = 0.039) frequency during baseline, together with a significantly higher endothelial NOi (*p* = 0.003) frequency during baseline recording. 

## 3. Discussion

Vasoconstriction is the primary reported response to hyperoxia, in spite of the contradictions which still exist [1,2,16]. Locally, O_2_ inhibits NO release, which would explain the vasoconstriction in most vascular beds. However, different responses have been registered without any convincing explanation. Cross talk among the different regulatory agents is a major determinant we need to better understand. Hyperoxemia seems to deactivate carotid body chemoreception, reducing sympathetic activity, which evokes a negative feedback between chemoreflex and baroreflex, such that inhibition of the carotid body triggers the baroreflex response [16,17]. Anesthesia is another major determinant to consider. Ketamine/xylazine anesthesia was suggested [18] to increase parasympathetic activity and suppress sympathetic and baroreceptor activity on rats, and a recently published study in the mouse shows that anesthesia definitively affects LDF component amplitudes associated to sympathetic activity and cardio-respiratory limits [15]. Finally, experimental design is a critical determinant. As we recently reported by distinct approaches, in human as in animal, vigil (human) or anesthetized (mouse) when perfusion changes in one of the limbs, circulation of both limbs cooperates to readjust to a new perfusion set-point [6,19]. Thus, distal (hind limb) measurements in vivo should always involve recordings from both limbs to reduce variability. The inclusion of male and female animals in the tested disease groups avoids any potential bias related with sex [20].

In the present study, a prevalent perD response was detected in all groups, as expected in all animals from Control group and the majority in DB and CH groups (Table 1). While some perMIX responses were consistently present, these had no statistical relevance. As suggested previously [6], this (perMIX) response might result from a regional adaptation to sudden perfusion changes but might also be an artifact from the LDF technique, known to be affected by significant variability. Keeping in mind that this apparent perfusion balance between limbs needs to be further studied, and that the main objective of this paper lies in reinforcing the analytical capacities of the hyperoxia model itself, we decided to exclude the perMIX responses from further analysis.

WA seems to confirm that flow motion has a universal nature, no matter the species, where the highest recorded frequency signals are attributed to heart and respiratory activities taken together. Mean heart and respiratory rates of vigil mice are 500–600 beats/ minute corresponding to 8.3–10 Hz, and 84 to 230 cycles / minute, or 1.4–3.8 Hz, respectively. Heart and respiratory rates from C57/BL6 mice under ketamine-xylazine anesthesia were 280 beats/ minute and 240 cycles/ minute, respectively (means), corresponding to 4.6 and 4.3 Hz. These very similar rates are not distinguishable in a frequency spectrum, and therefore overlap. We keep in mind that ketamine has a positive chronotropic effect, likely not disclosed by the negative chronotropic effect of xylazine [21]. The four lower frequency bands here described agree, in terms of frequency range, with the myogenic, sympathetic, NOd and NOi activities previously described for humans.

With this model, we recently demonstrated that perfusion is profoundly modified in the new HLI vessels [6]. We are currently further testing this model by applying the WA to detect vascular changes in diabetic and cardiac failure mice, as they cannot be detected by direct comparison of LDF perfusion curves. These two conditions are known to affect microcirculation in different ways. In human, diabetes affects primary organs and territories (retina, kidney, foot) microcirculation by multiple mechanisms, ultimately affecting endothelium vasodilation and reducing local reflex responses [22,23,24,25,26]. In turn, endothelial dysfunction and inflammation seem to be present in chronic heart failure patients, most likely associated with the ageing process and involving reduction of vascular reactivity [27,28].

WA shows that hyperoxia decreases the cardiorespiratory component activity in all groups, which return to their reference values in the recovery period except for the NHE1-OE group (Table 2). In the Control group hyperoxia also decreases myogenic activity, and significantly changes both NOd and NOi activities in the recovery period (Phase 3), likely because oxygen is a direct suppressor of the endothelial secretion activity (Table 2). These results agree with recently published data exploring a similar model [6].

In the DB group, db/+ mice have a recessive mutation with no phenotype described, being regarded as identical to Control group. In fact, hyperoxia evoked in db/+ and db/db animals a similar cardiorespiratory component changes as seen in the Control group. A similar reduction for the myogenic component in db/+ is also shown, while no other significant changes are noted, which seems to confirm that the behavior of db/+ is not very different from the Control healthy group. Interestingly, some significant differences are found for the myogenic (baseline and recovery after hyperoxia) and the endothelial components when compared with the Control group. These might be age-related differences or might be regarded as DB group markers, consistent with the known pathophysiology of the disease, where a decrease in microcirculatory vasomotion affects perfusion and local regulation [22]. The sympathetic overactivity noted for the db/db animals, although not significant, is also in line with the current knowledge on the pathogenesis of diabetes [23] (Table 2).

Regarding the CH group, NHE1-WT animals show that hyperoxia primarily reduces the cardiorespiratory component amplitude (*p* = 0.031), which then recovers to baseline values. Myogenic and NOi components are the other significant markers, as in the Control group, but in distinct phases. Although increasing during hyperoxia, a significant increase in the myogenic component amplitude is only seen in the recovery phase. Also interesting is the increase of the sympathetic component in all phases, although not statistically significant. In turn, the amplitudes are significantly higher in Phases 2 and 3 for NOi components, and have an opposite evolution for NOd components, relative to Control (Table 2). The comparison between NHE1-WT and Control components, obtained under these conditions, confirms that significant differences are mostly present in the baseline recordings, for cardiorespiratory, myogenic and endothelial components, and also for the recovery period of the cardiorespiratory component, likely linked to the background genotype and cardiac failure, being a good indicator for this analysis. Comparing both wild types, since db/+ is regarded as “normal”, significant differences are detected in the cardiorespiratory component in all phases, since this is likely the most expressive marker of NHE1 group (Table 2). In the NHE1-OE subgroup we note that hyperoxia significantly reduces the cardiorespiratory component frequency, as in the Control group. However, unlike the increase in the recovery phase for the Control group, these animals do not recover to baseline values during the experiment duration, showing instead a significant reduction of this component relative to baseline. This is likely related with their background and pathology, as which is a good indicator of analytical sensitivity (Table 2). This meets our expectations, since this transgenic strain overexpresses a protein that compromises the myocyte performance, producing cardiac remodeling and failure [29]. Also noteworthy is the decrease in the myogenic frequency (Phases 2 and 3), and the sustained increase in the sympathetic component activities. The increase in the cardiac sympathetic nerve activity and respective peripheral drive has been reported in cardiac hypertrophy [24], and in fact we note that this component behaves significantly different in these two subgroups at baseline (Table 2). Regarding the endothelial component we note the slight increase (Phase 2) and significant recovery (Phase 3) as in Control group for NOd frequencies. NHE1-OE and Control components, obtained under these conditions, confirm that significant differences are mostly present in the baseline and recovery periods for the cardiorespiratory and myogenic components. Comparing components frequencies from both NHE1 subgroups we confirm that significant differences between these groups are present at the baseline recording of sympathetic and endothelial (NOd and NOi) components (Table 2).

## 4. Materials and Methods

### 4.1. Animals

Forty-one animals were selected and assigned to different groups—a Control group with 8 C57/BL6 non-transgenic male mice (8 weeks old (w.o.), 22.7 ± 1.0 g); a spontaneous mutated diabetic group (DB) including 8 transgenic C57BLKsJ-db/db obese diabetic mice, 4 male and 4 female, (16.0 ± 3.2 w.o, 43.1 ± 12.7 g) and the corresponding internal controls of 8 age-matched C57BLKsJ-db/+ mice, 4 males and 4 females, (16.0 ± 3.2 w.o., 28.3 ± 12.7 g); and, a transgenic cardiac hypertrophy group (CH) with 9 FVB/NJ cardiac α-MHC-NHE-1 mice prone to develop cardiac failure, 7 males and 2 females, (20.7 ± 3.6 w.o., 29.7 ± 1.6 g) and the corresponding 8 age-matched internal controls, 6 males and 2 females, (23.4 ± 8.1 w.o., 29.6 ± 6.4 g). In this well-established model of obesity-induced type 2 diabetes [30], the db/db mice (homozygotes) carry a recessive mutation in the leptin receptor gene, which makes them resistant to its effects, including appetite suppression [31]. The db/+ mice (heterozygotes) have normal leptin receptor expression, no phenotype described. The FVB/NJ diseased mice overexpress the constitutively active mutant of sodium-proton exchanger 1 specifically in cardiac myocytes (NHE-OE), a protein mainly responsible for intracellular pH maintenance that produces cardiac hypertrophy and heart failure [29], while the internal controls have a normal endogenous NHE1 expression (NHE1-WT) (Table 3).

Type 2 obesity related diabetes was confirmed previously in a separate DB group composed of 6 C57BL/KsJ-db/db mice (3 males, 3 females, 12 w.o., 43 ± 3 g) with 320 ± 140 mg/dL glycemia measured by the FreeStyle papillon Vision System (Abbot, USA). Cardiac failure was confirmed in the CH group NHE1-OE mice by a reduced ejection fraction (<40%) measured by an echography imager (Visualsonics VEVO 2100 UK, with a 35 MHz probe MS400-0063).

Animals were installed at the INSERM U1034 animal facility, with controlled temperature and humidity conditions (21 ± 1 °C, 40–60%), exposed to regular 12 h light / 12h darkness cycles with food and water ad libitum. Animal experiments were performed in accordance with the guidelines from Directive 2010/63/EU of the European Parliament on the protection of animals used for scientific purposes, approved by the local Animal Care and Use Committee of Bordeaux University, complying with recently published Principles and standards for reporting animal experiments [19].

### 4.2. Experimental 

#### 4.2.1. Anesthesia and Setting

Animals were anesthetized by an intraperitoneal administration of a saline mixture of ketamine (125 mg/kg, Imalgene, Merial, Duluth, Georgia USA) and xylazine (10 mg/kg, Ronpum, Bayer, Munich, Germany), providing sedation for 50 min. Under anesthesia, animals were laid horizontally onto a surgical pad placed on top of an electric mat (Rainforest Heat Wave Mat Substrate Heater, Exo-Terra, Mansfield, MA, USA) kept at 36 °C. Their heads were fixed to the pad by a proper adapter, while the respective hind limbs were lateralized.

#### 4.2.2. Hyperoxia

The oxygen (O_2_) content was supplied by a suitable cylinder connected to the pad (AirLiquide, Bordeaux, France). For the hyperoxia challenge, oxygen was continuously administered (0.5 L/min) to ensure an inhalation fraction ≅ 100%.

Recordings were taken in three phases, each of ten minutes duration—a stabilization phase (Phase 1), with animals breathing the room atmosphere; a provocation phase (Phase 2), with animals breathing a 100% saturated oxygen atmosphere; and a recovery phase (Phase 3), returning to room atmosphere. As discussed ahead, exposure time to oxygen seems to be a critical determinant of the responses following. These timings were chosen based on our experience on human exposure to hyperoxia, as a similar response pattern was found in mice [5,16].

#### 4.2.3. Data Collection and Signal Analysis

Blood perfusion was primarily quantified by LDF, expressed in arbitrary blood perfusion units (AU). Two LDF probes were attached by double-sided adhesive strips (PF 105-3 Double-sided Tape Strips, Perimed, Järfälla, Sweden) to the inferior aspect of both paws, one probe for each paw, close to the posterior limit of plantar footpads. LDF signals were collected at a 32 Hz sampling frequency, using a time constant of 0.01 s from two PF 407 probes connected to two PF5010 modules of a Periflux 5000 system (Perimed).

**Figure 3:**
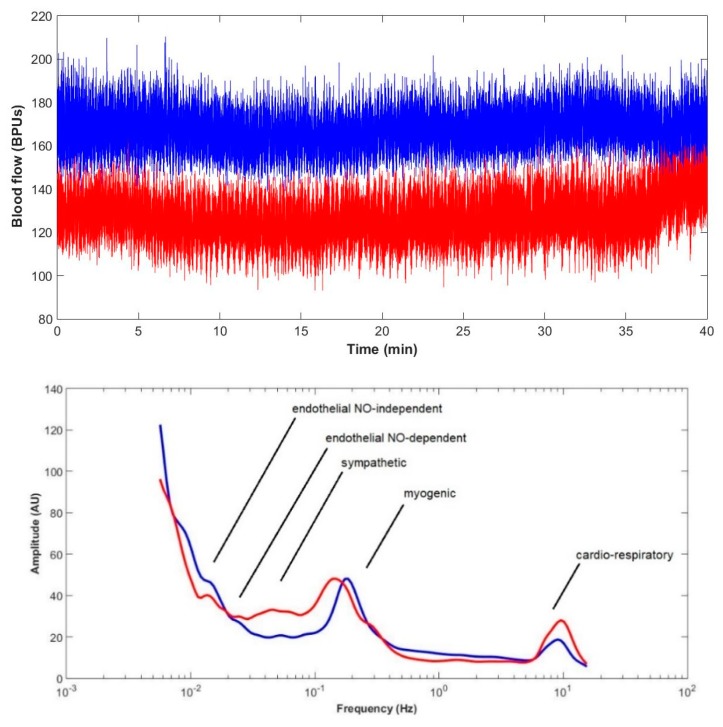
Baseline LDF signal registered in ta subset of the Control group animals for 40 min (**top**) and respective mean power spectrum obtained with the Wavelet analysis (WA) (**bottom**). Simultaneous recordings from both limbs (left, in red and right, in blue) are shown (*n* = 5).

A subset of the Control group (*n* = 5) was submitted to a 40 min baseline perfusion measurement in both hind limbs to obtain a clear spectral profile of the LDF signals for further processing (Figure 3). As shown, we obtained a similar profile to what is known in human, with the exception of the cardiac and respiratory bands, where a clear distinction between them could not be found. Instead we noted one single (merged) cardiorespiratory band.

The hyperoxia provocation procedure was equally applied to all groups time and compared. The vascular response to O_2_ provocation was evaluated by the perfusion changes in each phase and modelled with by the WA, as follows:

A wavelet is defined as a small wave or oscillation that decays quickly. Wavelets are considered a family of functions constructed from translations and dilations of a single function called the “mother wavelet” ψ(t). They are defined by:

(1) $$\psi_{a,b}\left(t\right)=\frac{1}{\sqrt{\left|a\right|}}\psi \left(\frac{t-b}{a}\right),\;a,b\in R,\;a\neq 0$$

The parameter a is the scaling parameter or scale, and it measures the degree of compression. The parameter b is the translation parameter which determines the time location of the wavelet. If |a| < 1, then the wavelet in the above equation is the compressed version (smaller support in time-domain) of the mother wavelet and corresponds mainly to higher frequencies. On the other hand, when |a| > 1, then $\psi_{a,b}\left(t\right)$ has a larger time-width than ψ(t) and corresponds to lower frequencies. Thus, wavelets have time-widths adapted to their frequencies [24]. WA allows the decomposition of a complex multiscaled signal into its main frequency components and the estimation of the contribution of each component to the overall signal in each time point [32].

#### 4.2.4. Statistics

Three periods, one of each phase of the protocol, were selected for statistical analysis—resting phase, considered between 6:30 to 9:30 min; the provocation phase, considered between 10:00 and 13:00 min, and the recovery phase, considered between 18:00 and 21:00 min. Descriptive (Microsoft Excel, USA) and nonparametric statistics (IBM SPSS Statistics, v21.0, IBM Corporation, Armonk, NY, USA) were calculated. After confirmation of the stability of the signal in a subset of Control group animals, the WA was applied to LDF raw signals using a MATLAB-based toolbox (Morlet wavelet function). This analysis, first proposed for the mouse by our group [33], allows the identification of dominant frequencies for each component.

The Wilcoxon signed-rank test was used for phase comparisons, while the Mann–Whitney test was used for comparisons between hind limbs (left versus right) on the same animal group and for inter-group (Control versus test animals), and a 95% confidence level adopted.

## 5. Conclusions

Our hyperoxia-mouse model complemented with the component analysis by the WA effectively provides a deeper observation to this study. The methodology seems to be reproducible and robust, considering that (a) it uses a key physiological player in microcirculatory homeostasis as a challenger, providing measurements as near as possible to the normal physiological conditions, (b) variability often attributed to the stressor, to the perfusion signal or to anesthesia, is dramatically reduced by using data from both limbs, avoiding readings in different moments of the perfusion adaptation following the challenge, (c) the WA component provides a very stable signal compared to the LDF raw signal, one that in the presence of oxygen leads to identifiable markers which, (d) can be applied to any other signal with the same pulsatile nature (e.g., photoplethysmography) for (e) both in human (vigil) or animal (under adequate anesthesia).

The model depicts a clear functional distinction for Control, DB, and CH animals, also between the expressed set and the respective (non-expressed) wild type. DB animals’ main markers were found in the cardiorespiratory, myogenic and endothelial components, in particular in the recovery phase for db/db mice. The main markers for CH animals (again cardiorespiratory, myogenic and endothelial components) were already different from Control in the baseline recordings, but clearly different at the recovery in the NHE1-OE mice. Also noteworthy was the increase of the sympathetic components (although not statistically significant) in the db/db set, and more obviously in the NHE1-OE animals, in both cases reported as a main component of these pathophysiological processes. We believe this is a positive indicator of the method’s sensitivity, considering that all animals were under the same anesthesia (see above).

The present experimental approach reduces variability, improves reproducibility and discriminative capacities, and provides a means to further explore more mechanistic views. Therefore, we recommend this hyperoxia-mouse model as an experimental challenger in vascular medicine.

## Figures and Tables

**Table 1:** Mean hind limb perfusion changes, expressed in BPUs (arbitrary blood perfusion units) registered in the left and right hind limb of each animal group during the experimental protocol (see text). Statistical comparison between hind limbs is shown in each phase of the protocol (m ± sd: mean ± standard deviation; * *p* < 0.05).

BPUs (m ± sd)/Groups	Phase 1		Phase 2		Phase 3	
	Right Limb	Left Limb	Right Limb	Left Limb	Right Limb	Left Limb
Control (*n* = 8)	120.5 ± 13.8	126.0 ± 25.1	101.2 ± 17.7	105.4 ± 29.8	127.7 ± 13.8	125.1 ± 38.61
*p*	0.943		0.943		0.724	
db/+ (*n* = 8)	139.6 ± 49.7	141.9 ± 41.1	129.4 ± 64.2	134.8 ± 52.6	132.8 ± 63.2	144.84 ± 9.1
*p*	0.645		0.574		0.505	
db/db (*n* = 8)	135.8 ± 47.4	127.0 ± 15.4	123.8 ± 50.8	130.2 ± 48.3	119.2 ± 71.4	114.1 ± 56.5
*p*	0.721		0.574		0.878	
NHE1-WT (*n* = 8)	158.1 ± 36.4	140.6 ± 34.3	138.2 ± 42.4	127.2 ± 33.0	145.7 ± 35.0	153.0 ± 36.9
*p*	0.234		0.645		0.505	
NHE1-OE (*n* = 9)	138.9 ± 29.8	128.2 ± 25.8	118.0 ± 25.0	106.8 ± 21.6	133.0 ± 29.5	113.7 ± 27.0
*p*	0.387		0.297		0.161	

**Table 2:** Mean frequency ranges and dominant (between brackets) frequencies in Hertz, obtained in all groups of tested animals (*n* = 41, animals (8 Controls, 8 db/+, 8 db/db, 8 NHE1-WT, 9 NHE1-OE) during the different phases of the experimental protocol (Ph1-baseline; Ph2-hyperoxia; Ph3-recovery). Frequencies are obtained from LDF signals from both hind limbs (means), which allows the identification of each component band (see text). Comparative statistics are shown, for each component’s dominant frequency in each group, regarding baseline recording (* statistically significant difference *p* < 0.05).

LDF Components/Groups	Cardiorespiratory			Myogenic			Sympathetic			Endothelial (NOd)			Endothelial (NOi)		
	Ph1	Ph2	Ph3	Ph1	Ph2	Ph3	Ph1	Ph2	Ph3	Ph1	Ph2	Ph3	Ph1	Ph2	Ph3
Control	5.400–3.200 (4.0)	4.700–2.300 (3.2)	6.000–3.4000 (4.4)	0.170–0.060 (0.1)	0.160–0.050 (0.085)	0.170–0.060 (0.17)	0.054–0.020 (0.031)	0.050–0.022 (0.031)	0.056–0.021 (0.037)	0.018–0.010 (0.014)	0.021–0.010 (0.014)	0.019–0.009 (0.016)	0.009–0.005 (0.007)	0.009–0.004 (0.006)	0.009–0.005 (0.008)
	* 0.005			* 0.002			0.972			0.363			0.624		
	* 0.049			0.221			0.075			* 0.005			* 0.025		
db/+	4.900–3.700 (4.1)	4.000–3.000 (3.3)	4.600–3.100 (3.9)	0.140–0.020 (0.079)	0.140–0.040 (0.071)	0.160–0.040 (0.086)	0.034–0.018 (0.029)	0.040–0.018 (0.027)	0.040–0.020 (0.029)	0.018–0.009 (0.015)	0.018–0.009 (0.013)	0.014–0.008 (0.013)	0.008–0.004 (0.0063)	0.009–0.004 (0.011)	0.008–0.004 (0.0069)
	* 0.001			* 0.023			0.346			0.666			0.132		
	* 0.019			0.975			0.683			0.806			0.074		
*p*-value Control versus db/+	0.202	0.202	0.867	* 0.022	0.061	* 0.019	0.830	0.202	0.061	0.185	0.756	0.325	* 0.038	0.141	0.350
db/db	4.700–3.000 (3.8)	4.200–3.000 (3.5)	5.300–3.200 (4.7)	0.120–0.050 (0.076)	0.140–0.050 (0.084)	0.150–0.050 (0.094)	0.040–0.017 (0.027)	0.050–0.021 (0.032)	0.040–0.014 (0.036)	0.016–0.009 (0.012)	0.021–0.010 (0.013)	0.020–0.010 (0.015)	0.009–0.005 (0.0078)	0.009–0.004 (0.0071)	0.009–0.005 (0.0072)
	* 0.023			0.701			0.152			0.346			0.213		
	* 0.003			0.576			0.433			1.000			0.061		
*p*-value Control versus db/db	0.840	0.125	0.264	* 0.001	0.169	* 0.003	0.264	0.840	0.064	0.579	0.614	* 0.010	0.687	0.336	* 0.039
*p*-value db/+ versus db/db	0.068	0.583	* 0.017	0.116	0.583	0.650	0.169	0.141	0.981	0.128	0.685	0.068	* 0.012	0.458	0.488
NHE1-WT	4.600–2.600 (3.2)	3.800–1.700 (3.0)	3.500–2.300 (3.2)	0.120–0.040 (0.077)	0.120–0.045 (0.08)	0.185–0.056 (0.105)	0.039–0.022 (0.032)	0.045–0.024 (0.033)	0.052–0.020 (0.035)	0.022–0.011 (0.015)	0.024–0.013 (0.014)	0.014–0.008 (0.013)	0.008–0.004 (0.0063)	0.009–0.004 (0.011)	0.008–0.004 (0.0069)
	* 0.031			0.475			0.894			0.126			* 0.037		
	0.118			* 0.041			0.455			0.824			* 0.049		
*p*-value Control versus NHE1-WT	* 0.001	0.168	* 0.001	* <0.001	0.110	0.406	0.936	0.689	0.810	* 0.046	0.936	0.470	* 0.003	0.137	0.225
NHE1-OE	4.500–2.700 (3.3)	3.700–2.000 (2.9)	3.500–2.400 (3.0)	0.120–0.040 (0.089)	0.109–0.045 (0.08)	0.110–0.042 (0.085)	0.038–0.019 (0.027)	0.026–0.013 (0.031)	0.033–0.025 (0.031)	0.016–0.008 (0.012)	0.011–0.007 (0.013)	0.020–0.012 (0.015)	0.011–0.007 (0.0085)	0.013–0.007 (0.0071)	0.012–0.007 (0.0073)
	* 0.044			0.133			0.507			0.887			0.485		
	* 0.002			0.492			0.078			* 0.039			0.102		
*p*-value Control versus NHE1-OE	* 0.001	0.170	* <0.001	* 0.016	0.082	* <0.001	0.183	0.489	0.062	0.798	0.417	0.293	0.650	0.859	0.441
*p*-value NHE1-WT versus NHE1-OE	1.000	0.744	0.072	0.465	0.950	0.200	* 0.035	0.305	0.368	* 0.039	0.104	0.755	* 0.003	0.072	0.632
*p*-value db/+ versus NHE1-WT	* <0.001	* 0.02	* <0.001	0.131	0.820	0.432	0.560	0.106	0.322	0.462	0.274	0.860	0.274	0.899	0.940

**Table 3:** Summary of the genetic profile of each selected group of animals regarding background, mutation and sex (see text).

	Name	Background	Mutation	Male	Female	Total
Control group	-	C57Bl/6	-	8	-	8
Control CH	NHE1-WT	FVB/NJ	-	6	2	8
CH group	NHE1-OE	FV/NJ	NHE1 overexpression	7	2	9
Control DB	Db/+	C57BL/KsJ	Lepr +/db	4	4	8
DB group	Db/db	C57BL/KsJ	Lepr db/db	4	4	8
